# Doctors’ alertness, contentedness and calmness before and after night shifts: a latent profile analysis

**DOI:** 10.1186/s12960-023-00855-z

**Published:** 2023-08-21

**Authors:** Maarten P. M. Debets, Fokkedien H. M. P. Tummers, Milou E. W. M. Silkens, Coen R. H. Huizinga, Kiki M. J. M. H. Lombarts, Koen E. A. van der Bogt

**Affiliations:** 1 Research Group Professional Performance and Compassionate Care, Department of Medical Psychology, Amsterdam University Medical Centres, University of Amsterdam, Meibergdreef 9, 1105 AZ Amsterdam, The Netherlands; 2 Amsterdam Public Health Research Institute, Amsterdam, The Netherlands; 3 Department of Health Services Research and Management, City University of London, London, United Kingdom; 4 Centre for Human Drug Research, Leiden, The Netherlands; 5 Department of Gyneacology, Leiden University Medical Centre, Leiden, The Netherlands; 6 Department of Surgery, Leiden University Medical Centre, Leiden, The Netherlands; 7 Department of Surgery, Haaglanden Medical Centre, The Hague, The Netherlands; 8 University Vascular Centre Leiden, The Hague, The Netherlands

**Keywords:** Doctors, Night shifts, Latent profile analysis, Well-being, Performance

## Abstract

**Background:**

While night shifts are crucial for patient care, they threaten doctors’ well-being and performance. Knowledge of how the impact of night shifts differs for doctors is needed to attenuate the adverse effects of night shifts**.** This study aimed to obtain more precise insight into doctors’ feelings surrounding night shift by: identifying profiles based on doctors’ alertness, contentedness and calmness scores before and after night shifts (research question (RQ) 1); assessing how doctors’ pre- and post-shift profiles change (RQ2); and determining associations of doctors’ demographics and shift circumstances with alertness, contentedness and calmness change (RQ3).

**Methods:**

Latent Profile Analysis using doctors’ pre- and post-shift self-rated alertness, contentedness and calmness scores was employed to identify pre- and post-shift profiles (RQ1). A cross-tabulation revealed pre- and post-shift profile changes (RQ2). Multiple regressions determined associations of demographics (i.e. age, sex, specialty) and night shift circumstances (i.e. hours worked pre-call, hours awake pre-call, shift duration, number of consecutive shifts, total hours of sleep) with alertness, contentedness and calmness change (RQ3).

**Results:**

In total, 211 doctors participated with a mean age of 39.8 ± 10 years; 47.4% was male. The participants included consultants (46.4%) and trainees (53.6%) of the specialties surgery (64.5%) and obstetrics/gynaecology (35.5%). Three pre-shift (Indifferent, Ready, Engaged) and four post-shift profiles (Lethargic, Tired but satisfied, Excited, Mindful) were found. Most doctors changed from Ready to Tired but satisfied, with alertness reducing most. Age, specialty, sleep, shift duration and the number of consecutive shifts associated with alertness, contentedness and calmness changes.

**Conclusions:**

The results provided nuanced insight into doctors’ feelings before and after night shifts. Future research may assess whether specific subgroups benefit from tailored interventions.

**Supplementary Information:**

The online version contains supplementary material available at 10.1186/s12960-023-00855-z.

## Introduction

Providing high-quality patient care inevitably involves working night shifts for most doctors. Night shifts can negatively impact doctors' health and performance [1–5]. Doctors that regularly work at night are more prone to chronic diseases [2], sleeping disorders [3], and burnout [5]. Due to sleep loss and circadian misalignment, shift work also associates with fatigue, reduced alertness and impaired psychomotor and cognitive performance [6–11]. Consequently, doctors that regularly work night shifts are more likely to make significant medical errors due to the effects of these shifts [5, 12]. Therefore, it is crucial for doctors and patients that night shifts' adverse effects are maximally attenuated.

The first steps to attenuate these adverse effects have been made. Internationally, regulating authorities such as the Accreditation Council for Graduate Medical Education (ACGME) restricted duty hours to protect doctors and patients. While some researchers report that general interventions like reducing working hours seem beneficial [13], others did not find effects on patient outcomes or doctors’ well-being [14]. Even with reduced working hours, doctors’ may still feel burned out and experience a suboptimal quality of life due to working at night and, as a result, intend to leave the profession [15]. More detailed research on doctors’ feelings surrounding night shift is needed to attenuate its adverse effects further and optimise patient care.

This study focuses on doctors’ self-rated alertness, contentedness (mood) and calmness before and after night shifts, which is important for several reasons. Firstly, subjective assessments are likely to influence behaviour and decision making [16]. For example, the decision to drive home after a night shift is likely to be made based on perceptions of alertness, which does not robustly relate to objective driving performance [17]. Another example is that doctors who rated their mood as bad indicated that they talk less with patients than their peers in good moods [18]. Secondly, to prevent severe well-being issues like burnout, it is necessary to know how night shifts affect doctors contentedness. Feeling discontented can be a timely signal that one needs to recover and not engage in challenging activities [5, 19]. Lastly, based on doctors’ varying levels of alertness, contentedness and calmness, efforts to reduce night shifts’ adverse effects can be customised and gain effectiveness, complementing general interventions.

Several studies show that night shifts can negatively impact doctors’ alertness and various mood states related to contentedness and calmness [5, 6, 8, 9, 20–23]. These studies also show that sleep, the shifts’ duration and the number of consecutive shifts are crucial for doctors’ performance and well-being [5, 6, 8, 9, 20–23]. However, it is unknown how the impact of night shifts differs for individuals. Person-centred analyses, such as latent profile analysis, are suitable for identifying specific subgroups and complement existing studies by providing more insight into the heterogeneity of doctors' alertness, contentedness, and calmness surrounding night shifts [24]. Researchers recommend using knowledge obtained by person-centred analyses to customise interventions [24]. In addition, more information on how night shift circumstances differ between subgroups and which shift circumstances predict alertness, contentedness and calmness change would be informative to shape interventions. However, such knowledge is lacking, hampering the design of tailor-made interventions to attenuate the adverse effects of night shifts. Therefore, this study will answer the following research questions (RQ):

RQ1: What profiles can be identified based on doctors’ alertness, contentedness and calmness scores before and after night shifts?

RQ2: Do doctors’ profiles and respective alertness, contentedness and calmness scores before the night shift differ from after the night shift and if so, how?

RQ3: Which doctor demographics and night shift circumstances associate with changes in alertness, contentedness and calmness?

## Methods

### Setting

In the Netherlands, night shifts are organised differently for consultants and trainees. Trainees are in-house for a maximum of 12 h during the night. They have protected time off after a night shift and usually perform multiple consecutive shifts. Consultants supervise trainees and work only on demand during the night. The scheduling of consultant shifts may vary per hospital. Most consultants perform on-call night shifts following their regular day shift. Some consultants are scheduled to continue to work immediately following their night shift, performing another regular day shift, while others have a protected day off.

### Participants and data collection

This project is part of a larger Dutch research project on fitness to perform, for which a comprehensive description of data collection is published by Tummers et al. [25, 26]. The project collected data on doctors’ objective performance and their subjective alertness, contentedness and calmness scores the latter of which were used in this study. This study’s participants were consultants or trainees from the specialties surgery and obstetrics and gynaecology (OB/GYN) from nine Dutch hospitals. They rated their alertness, contentedness and calmness levels before and/or after one or more night shifts using a questionnaire. Trainees completed the pre-call measurements before the start of the night shift, usually around 10 PM. Consultants completed pre-call measurements around the transition from dayshift to being on-call for the night (around 5 PM). For all doctors, post-call measurements were completed at the change from night shift to dayshift (around 8 AM). Participants were asked to complete the pre- and post-call measurements within 1 h before or after the change of shifts.

### Measurements

Participants completed the 16-item Bond-Lader questionnaire measuring 3 domains: alertness (nine items), contentedness (five items) and calmness (two items) [27]. Table 2 in the results section provides an overview of the composite scale scores and the individual items scores. Each item was scored using a visual analogue scale (VAS), which means that answer scales were depicted using bipolar 100 mm horizontal lines with two opposing adjectives at either end (e.g. Alert-Drowsy, Contented-Discontented, Calm-Excited). The Bond-Lader questionnaire has established validity in clinical practice and relevant reference frames [27, 28]. For example, the legal driving limit of 0.06% ethanol intoxication (i.e. two units of alcohol) corresponds with − 8.17 points on alertness [28]. Doctors’ demographics and night shift circumstances were assessed by an additional questionnaire (Additional file 1). Demographics included in this study were: sex (male/female), age in years, clinical function (consultant/trainee), specialty (surgery / OB/GYN), average hours of sleep per night, and average hours of work per week. Included night shift circumstances were: hours worked before the night shift, hours awake before the night shift, shift duration in hours, number of consecutive shifts, hours awake, hours of sleep during the night shift, and activity during the night shift (% rest, operating room (OR), emergency room (ER), telephone).

### Statistical analyses

Doctors with missing data on subjective alertness, contentedness and calmness were excluded. To answer our research questions, we divided our total sample into three separate samples: (1) all doctors with a pre-shift measurement, (2) all doctors with a post-shift measurement and (3) doctors with paired measurements only. The pre and post-shift samples hold unique data that are not represented in the paired sample, but samples do partly overlap as doctors with paired measurements were also incorporated in the pre- and post-shift samples. For RQ1 (identifying pre- and post-shift profiles) we used the pre- and post-shift samples. Using the pre- and post-shift samples guaranteed that we calculated profiles on the most complete data and therefore represented reality closest. Furthermore, we chose to analyse the unequal pre- and post-shift samples to have the largest sample of unique doctors as possible given the data. For RQ2 (assessing pre- and post-shift profile changes) and RQ3 (associations of demographics and night shift circumstances with alertness, contentedness and calmness change) we used the sample with paired measurements. For doctors with measurements from multiple shifts, we only included the first paired, pre- or post-shift measurement to assure data independence and equal weighting of participants. Descriptive statistics were used to summarise the characteristics of doctors in each sample. As the samples were not completely identical, ANOVA and χ^2^ were used to analyse differences in doctors’ demographic characteristics.

To answer RQ1, we calculated doctor profiles separately for the pre- and post-shift sample using Latent Profile Analysis (LPA). LPA is a statistical modelling approach that tries to identify groups of individuals (i.e. latent profiles) based on their responses to a series of continuous variables [29]. LPA is a person-centred analysis that can reveal hypothetical patterns in the data and find the ‘less obvious’ subgroups [24]. It complements variable-centred analyses, which look for associations between variables for the entire sample or subgroups made based on demographic variables [24, 29]. Previous studies have used this method to identify burnout phenotypes among surgery trainees [30], patterns in unprofessional behaviours of medical students [31], or classify clinical departments’ learning climate performance [32]. In this study, LPA was conducted in the R environment (version 3.6.1.) using the MClust 5.4.6. package. Profiles were calculated using standardised domain scores of alertness, contentedness and calmness.

The following three criteria were used to determine the most optimal profile solution: (1) the model with the highest Bayesian Information Criterion (BIC) was selected, (2) the selected model was specified using Bootstrap Likelihood Ratio Testing (BLRT) to compare the increase in model fit when an additional profile was added using the *p*-value provided by this method (e.g. 2 vs 3 profiles *p* = 0.999), and (3) the three best profile solutions were discussed and interpreted by the research team (profile interpretability) [33]. Based on the selected profile solution, each respondent was assigned to a pre- and post-shift profile in R. All further analyses were conducted in IBM SPSS Statistics for Windows, version 26 (IBM Corp., Armonk, N.Y., USA).

Both consultants and trainees were included in the LPA because we expected differences in night shift circumstances to become apparent when comparing the different profiles. Such an approach may provide more nuanced insight into the differences between how consultants and trainees perceive their night shift than a priori dividing them into separate groups. Descriptive statistics were used to calculate doctors’ alertness, contentedness, and calmness scores within the identified pre- and post-shift profiles. Descriptive statistics were also used to describe doctors and their night shift circumstances within each profile. ANOVA and χ^2^ tests assessed differences between the profiles. For the ANOVA, results were checked for consistency using Brown-Forsythe and Welch F-test given potential unequal sample sizes and variances within profiles as these tests are more robust to such violations. Bonferroni and Games-Howell post-hoc tests were performed, both correcting for multiple comparisons. Fisher’s exact test was used instead of χ^2^ when data had more than 20% of less than 5 counts.

To answer RQ2, we conducted a before and after the night shift measurement. We selected doctors with paired measurements and identified their pre- and post-shift profiles using a cross-tabulation. The pre-shift profiles were entered as rows and the post-shift profiles as columns to identify how doctors’ profiles changed. We inspected the corresponding change in alertness, contentedness, and calmness scores for each observed combination of pre- and post-shift profiles.

For RQ3, we further investigated the sample with paired measurements to determine which demographic variables and night shift circumstances were predictive of changes in alertness, contentedness and calmness. To that end, we conducted multiple regressions on the change scores of each outcome variable (post-shift minus pre-shift scores). Negative regression coefficients indicate deterioration in the corresponding outcome. Demographics included in the regression were age, sex, and specialty. Included night shift circumstances were hours worked pre-call, hours awake pre-call, shift duration, number of consecutive shifts, and total hours of sleep. The relative importance of the tested effects was assessed using Cohen’s guidelines, which state that a β of 0.10–0.29 is considered a small effect, 0.30–0.49 a moderate effect and 0.50 or greater a large effect [34, 35].

Boxplots revealed two outliers on calmness; one in the pre-shift sample (score = 21.50) and one in the post-shift sample (score = 9.00), from different respondents. The research team discussed these cases and concluded they represent realistic values within the scoring range of 0 to 100. Since these values were expected to occur in the population too, they were included in the analyses.

## Results

### Study participants

This study’s total sample comprised 211 doctors with a mean age of 39.8 ± 10 years; 47.4% was male. The sample included consultants (46.4%) and trainees (53.6%) of the specialties surgery (64.5%) and OB/GYN (35.5%). Table 1 describes the study participants within each subsample. ANOVA and χ^2^ showed no substantial differences between the samples. Table 2 presents he alertness, contentedness and calmness of doctors within each sample.

**Table 1:** Participant characteristics by sample

	Pre (N = 189)	Post (N = 157)	Paired (N = 135)
Age M ± SD Missing#	40.4 ± 9.95 1	39.3 ± 9.82 1	40.1 ± 9.77 1
Male n(%)	92 (48.7%)	73 (46.5%)	65 (48.1%)
Female n(%)	97(51.3%)	84 (53.5%)	70 (51.9%)
Consultants n(%)	95 (50.3%)	71 (45.2%)	68 (50.4%)
Trainees n(%)	94 (49.7%)	86 (54.8%)	67 (49.6%)
Surgery n(%)	115(60.8%)	97(61.8%)	76 (56.3%)
OB/GYN n(%)	74(39.2%)	60 (38.2%)	59 (43.7%)
Avg. sleep M ± SD Missing#	6.82 ± .72 17	6.76 ± .70 12	6.80 ± .72 11
Avg. work M ± SD Missing^#^	50.5 ± 8.72 29	50.3 ± 8.08 23	49.4 ± 7.89 20

ANOVA and χ^2^ for showed no significant differences in demographics between the samples (all *p-values* > .05). M = mean, SD = standard deviation

^#^Participants with missing data in demographics or descriptive variables are represented in the total N presented in this table

**Table 2:** Bond and Lader visual analogue scales (VAS)

Code	Item	Pre sample	Post sample	Paired sample (pre)	Paired sample (post)
Alertness	Composite score	69.3 ± 14.7	58.8 ± 18.9	71.4 ± 14.3	59.0 ± 18.7
VASBL01	Alert–Drowsy	29.2 ± 18.1	42.4 ± 22.1	26.3 ± 16.9	42.0 ± 21.7
VASBL03	Strong–Feeble	30.5 ± 16.6	39.3 ± 19.9	28.2 ± 15.4	39.3 ± 19.4
VASBL04	Confused–Clear Headed	70.6 ± 17.3	59.6 ± 22.0	72.9 ± 16.2	59.6 ± 21.6
VASBL05	Well-coordinated–Clumsy	26.5 ± 16.0	37.0 ± 20.3	24.6 ± 15.2	36.4 ± 19.6
VASBL06	Lethargic–Energetic	59.8 ± 19.3	45.9 ± 25.5	62.1 ± 18.9	46.3 ± 25.5
VASBL09	Mentally slow–quick-Witted	65.7 ± 19.0	54.2 ± 23.6	67.6 ± 18.2	53.7 ± 23.2
VASBL11	Attentive–Dreamy	32.0 ± 18.3	44.1 ± 22.0	30.2 ± 18.5	43.7 ± 22.0
VASBL12	Incompetent–Proficient	75.4 ± 15.1	66.1 ± 18.8	77.3 ± 14.1	65.9 ± 18.6
VASBL15	Interested–Bored	29.6 ± 17.7	33.6 ± 18.4	27.7 ± 16.8	33.6 ± 17.8
Contentedness	Composite score	72.0 ± 14.4	69.9 ± 15.5	73.7 ± 13.9	70.1 ± 15.3
VASBL07	Contented–Discontented	27.6 ± 16.3	28.3 ± 17.3	25.4 ± 15.4	27.9 ± 17.1
VASBL08	Troubled–Tranquil	70.7 ± 16.6	69.4 ± 19.3	72.1 ± 16.1	70.1 ± 18.3
VASBL13	Happy–Sad	26.0 ± 17.7	27.4 ± 16.8	24.3 ± 16.8	26.9 ± 16.2
VASBL14	Antagonistic–Amicable	74.0 ± 17.0	71.1 ± 17.4	75.5 ± 16.2	71.3 ± 17.0
VASBL16	Withdrawn–Gregarious	68.9 ± 18.0	64.5 ± 20.3	70.6 ± 17.7	63.8 ± 20.3
Calmness	Composite score	68.6 ± 17.3	68.6 ± 16.7	68.9 ± 17.9	69.8 ± 15.6
VASBL02	Calm–Excited	30.1 ± 19.5	28.9 ± 18.9	30.3 ± 20.6	27.9 ± 17.5
VASBL10	Tense–Relaxed	67.2 ± 18.8	66.1 ± 19.1	68.2 ± 18.9	67.5 ± 17.7

Score range is 0–100 from left to right, e.g., Alert (0)–Drowsy (100). The fomulea to calculate the composite alertness, contentedness, and calmness scores is provided in the Additional file 1: A

### Doctors pre- and post-shift profiles (RQ1)

Three pre-shift profiles (BIC = − 1349.75, Variable volume/Variable shape/Equal orientation (VVE) model; BLRT = 3 vs. 4 p = 0.945) and four post-shift profiles (BIC = − 1089.52, Variable volume/Equal shape/Equal orientation (VEE) model; BLRT = 4 vs 5 p = 0.165) were found (Table 3). We labelled the three pre-shift profiles as *Indifferent* (*n* = 35), *Ready* (*n* = 90), and *Engaged* (*n* = 64). On average, doctors in *Indifferent* were neither alert nor drowsy, contented or discontented, nor calm or excited. They scored neutral on all measures. Those in *Ready* were characterised by feeling somewhat alert, contented and calm. Doctors in *Engaged* reported high levels of alertness, contentedness and calmness.

**Table 3:** Doctors’ mood profiles before and after night shifts

	Name given	N =	Alert M ± SD	Content M ± SD	Calm M ± SD	Mixing probabilities
*Pre profile*						
1	Indifferent	35	52.6 ± 6.17	54.8 ± 5.64	51.7 ± 11.1	17.5%
2	Ready	90	67.1 ± 13.6	71.6 ± 13.3	65.4 ± 16.6	51.2%
3	Engaged	64	81.5 ± 6.95	82.1 ± 9.00	82.2 ± 8.78	31.3%
*Post profile*						
1	Lethargic	14	48.7 ± 4.25^a^	50.5 ± 2.96	49.9 ± 2.70^e^	7.7%
2	Tired but satisfied	91	48.1 ± 12.7^a^	64.6 ± 12.6^c^	66.5 ± 11.6	59.8%
3	Excited	11	72.0 ± 14.6^b^	75.7 ± 12.8^c,d^	40.6 ± 12.7^e^	7.8%
4	Mindful	41	82.5 ± 7.51^b^	86.5 ± 6.62^d^	87.1 ± 6.85	24.7%

Identical superscript letters indicate that these profile scores are NOT significant at *p* < 0.05 (equal variances not assumed). When equal variances are assumed all scores are significantly different

The four post-shift profiles were: *Lethargic* (n = 14), *Tired but satisfied* (*n* = 91), *Excited* (*n* = 11) and *Mindful* (*n* = 41). Doctors in *Lethargic* scored lower on all mood measures than the pre-shift profile *Indifferent*. Their scores slightly tended towards drowsiness and mental slowness, and tension. They were neither contented nor discontented. Doctors in *Tired but satisfied* had the lowest levels of alertness of all post-profiles, although they reported feeling somewhat contented and calm. Those in *Excited* were relatively alert and contented and showed low levels of calmness. Finally, doctors in *Mindful* showed relatively high alertness levels and even higher scores on contentedness and calmness.

Table 4 shows and compares doctors’ demographics and night shift circumstances per profile. Before the night shift, only the proportion of consultants and the age of doctors in the profiles *Ready* and *Engaged* differed significantly. The post-shift profiles showed more marked differences, especially between *Tired but Satisfied* and *Mindful*.

**Table 4:** Demographics and night shift circumstances per profiles

Pre-shift (N = 189)	1. Indifferent (N = 35)	2. Ready (N = 90)	3. Engaged (N = 64)	
Age M ± SD	39.5 ± 9.56	38.5 ± 9.57^3^	43.7 ± 9.98^2^	
Male n(%)	12 (34.3%)	43 (47.8%)	37 (57.8%)	
Female n(%)	23 (65.7%)	47 (52.2%)	27 (42.2%)	
Consultants n(%)	17 (48.6%)	34 (37.8%)^3^	44 (68.8%)^2^	
Trainees n(%)	18 (51.4%)	56 (62.2%)^3^	20 (31.3%)^2^	
Surgery n(%)	21 (60.0%)	58 (64.4%)	36 (56.3%)	
OB/GYN n(%)	14 (40.0%)	32 (35.6%)	28 (43.8%)	
Avg. sleep M ± SD	6.76 ± .66	6.87 ± .70	6.79 ± .77	
Avg. work M ± SD	49.2 ± 6.77	50.4 ± 8.96	51.3 ± 9.29	
Hrs. awake	9.73 ± 4.17	9.14 ± 4.44	9.60 ± 3.83	
Hrs. worked	4.80 ± 4.73	3.89 ± 4.72^3^	6.06 ± 4.85^2^	
Nmbr. cons shifts M ± SD	1.69 ± 1.35	2.17 ± 1.87	1.62 ± 1.45	

Post-shift (N = 157)	1. Lethargic (N = 14)	2. Tired but satisfied (N = 91)	3. Excited (N = 11)	4. Mindful (N = 41)
Age M ± SD	39.0 ± 9.63	36.4 ± 7.82^4^	33.6 ± 5.04^4^	47.2 ± 10.5^2,3^
Male n(%)	5 (35.7%)	37 (40.7%)^4^	3 (27.3%)	28 (68.3%)^2^
Female	9 (64.3%)	54 (59.3%)^4^	8 (72.7%)	13 (31.7%)^2^
Consultants n(%)	7 (50.0%)	29 (31.9%)^4^	1 (9.09%)^4^	34 (82.9%)^2,3^
Trainees n(%)	7 (50.0%)	62 (68.1%)^4^	10 (90.9%)^4^	7 (17.1%)^2,3^
Surgery n(%)	10 (71.4%)	51 (55.6%)	7 (63.6%)	29 (70.7%)
OB/GYN n(%)	4 (28.6%)	40 (44.4%)	4 (36.4%)	12 (29.3%)
Avg. sleep M ± SD	6.77 ± .53	6.80 ± .66	6.47 ± .71	6.75 ± .83
Avg. work M ± SD	50.5 ± 8.43	49.3 ± 8.24	48.9 ± 7.56	52.9 ± 7.45
(Pre)Hrs. awake M ± SD	8.85 ± 4.26	8.78 ± 4.22	7.20 ± 4.66	9.58 ± 3.87
(Pre)Hrs. worked M ± SD	4.54 ± 5.00	3.28 ± 4.64^4^	.60 ± 1.34^4^	7.36 ± 4.00^2,3^
Nmbr. cons shifts M ± SD	2.60 ± 2.41	2.30 ± 1.84^4^	2.80 ± 1.93	1.23 ± .77^2^
Duration shift M ± SD*	15.4 ± 6.26	13.4 ± 5.15^4^	12.0 ± 2.75	16.2 ± 5.46^2^
Hrs. sleep M ± SD	2.75 ± 2.80	1.83 ± 2.07^4^	2.10 ± 2.28^4^	4.83 ± 2.33^2,3^
Hrs. awake M ± SD	9.10 ± 8.91	10.9 ± 7.93^4^	11.0 ± 9.06	5.20 ± 7.60^2^
% Activity M ± SD				
Telephone	12.3% ± 11.0	17.6% ± 18.3	24.2% ± 24.0	12.5% ± 17.5
OR	17.0% ± 30.2	11.2% ± 17.6	16.7% ± 29.4	16.8% ± 23.3
ER	28.5% ± 28.5	43.1% ± 29.4^4^	40.2% ± 25.5	17.9% ± 23.1^2^
Rest	42.2% ± 33.4	28.1% ± 28.8^4^	18.9% ± 26.3^4^	52.9% ± 35.0^2,3^

Each superscript indicates the profile number from which differences are significant at *p* < .05. * *p* = .51. Pre-shift missings on demographic variables are: Age (n = 1), Avg. Sleep (n = 17), Avg. Work (n = 29), Nmbr. Consecutive shifts (n = 6), Hrs. awake (n = 6), Hrs. worked (n = 4). Post-shift missings on demographic variables are: Age (n = 1), Duration shift (n = 18), Nmbr. Consecutive shifts (n = 18), Hrs. sleep (n = 18), Hrs. awake (n = 18), Telephone (n = 18), OR (n = 18), ER (n = 18), Rest (n = 18)

### Profile changes before and after night shifts (RQ2)

**Fig. 1:**
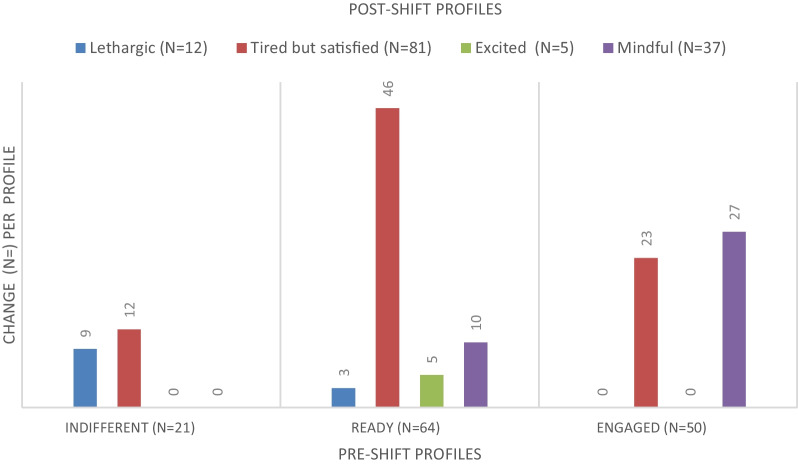
Descriptive data illustrating how doctors’ pre-shift profiles differ from their post-shift profiles (paired data)

Figure 1 shows to which profile doctors with paired measurements (*n* = 135) belonged before and after one night shift. Doctors from *Indifferent* were classified as *Lethargic* (*n* = 9) or *Tired but satisfied* (*n* = 12) after the night shift. No doctors from *Indifferent* changed to *Excited* or *Mindful*. Three doctors from *Ready* fell into *Lethargic*. The most observed profile change within *Ready* was to *Tired but satisfied* (*n* = 46). Five doctors from *Ready* moved to *Excited* and ten to *Mindful*. No doctors from *Engaged* were classified as *Lethargic* or *Excited* after the night shift. Instead, they fell into the profiles *Tired but satisfied* (*n* = 23) or *Mindful* (*n* = 27).

Additional file 1 shows the mean difference (post-shift minus pre-shift scores) of alertness, contentedness and calmness for each observed profile change. Doctors who changed from *Ready* to *Tired but satisfied*, the most observed switch, showed substantially reduced alertness (− 20.8 ± 15.96), decreased contentedness (− 5.76 ± 13.5) and improved calmness (+ 3.11 ± 21.5).

### Predictors of change in alertness, contentedness and calmness (RQ3)

Age (β = 0.262 (small effect); 95% CI 0.060 – 0.844; *p* = 0.024) was positively related to a change in alertness, meaning older doctors felt more alert after working a night shift. Doctors from the specialty OB/GYN decreased more on alertness than those from surgery (β = − 0.345 (moderate effect); 95% CI − 17.251 to − 5.653; *p* < 0.001). Shift duration (β = − 0.285 (small effect); 95% CI − 1.495 to − 0.200; *p* = 0.011) associated negatively with a change in alertness and total hours of sleep associated positively (β = 0.330 (moderate effect); 95% CI 0.667–0.3422; *p* = 0.004). The contentedness of doctors from OB/GYN reduced more than those from surgery (β = − 0.206 (small effect); 95% CI − 10.285 to − 0.189; *p* = 0.042). The number of consecutive shifts associated negatively with contentedness (β = − 0.302 (moderate effect); 95% CI − 3.718 to − 0.531; *p* = 0.009) and calmness change (β = − 0.307 (moderate effect); 95% CI − 5.509 to − 0.603; *p* = 0.015).

## Discussion

For surgery and OB/GYN consultants and trainees working night shifts, this study investigated their levels of alertness, contentedness and calmness and identified three pre-shift profiles (*Indifferent*, *Ready*, *Engaged*) and four post-shift profiles (*Lethargic*, *Tired but satisfied*, *Excited*, *Mindful*) (RQ1). Most doctors changed from *Ready* to *Tired but satisfied*, with the most substantial reduction in the realm of alertness. No movement was observed from *Indifferent* to *Excited* or *Mindful* and from *Engaged* to *Lethargic* or *Excited* (RQ2). The alertness of older doctors was less affected by the night shift. Compared to surgery, those from OB/GYN scored lower on alertness and contentedness. Doctors that slept more and had shorter shifts felt more alert, and having more consecutive shifts was detrimental to their contentedness and calmness (RQ3).

When comparing the identified pre- and post-shift profiles, it seems that doctors’ alertness, contentedness and calmness show more complex manifestations after the night shift. The pre-shift profiles distinguish themselves on relatively low, average and high scores on all three outcome measures. After the night shift, two profiles, namely *Lethargic* and *Mindful, also* represent doctors with the lowest and highest scores. In contrast to the pre-shift profiles, an additional post-shift profile was identified, and the scores within the post-profiles varied more from each other. This variation can be observed most clearly within the profiles *Tired but satisfied* and *Excited*. In *Tired but satisfied* doctors’ alertness scores were substantially lower than their contentedness and calmness levels. In *Excited*, low levels of calmness contradicted with relatively high alertness and contentedness scores.

Our findings suggest that measured demographics and night shift circumstances can only partially explain the differences in the identified profiles. Some differences between *Ready* and *Engaged* seem to originate from the unequal proportion of included consultants and trainees and their respective working circumstances. Consultants will usually be more experienced in performing night shifts and work more hours beforehand. Post-shift differences between *Tired but Satisfied* and *Engaged* and, to a lesser extent, *Excited* and *Engaged* could also be partly explained by included proportions of trainees and consultants and their respective night shift circumstances, such as sleep and the number of consecutive shifts. Perhaps more interesting is that the *Indifferent* and the *Lethargic* profiles did not significantly differ on demographics and night shift circumstances from the other profiles. This finding suggests that factors outside work should also be considered to safeguard or enhance doctors’ fitness to perform at night.

Multiple factors could explain the obtained profiles, such as doctors’ individual preferences or personal situation [36, 37], genetics in terms of needing sleep [38], or unmeasured working circumstances such as experienced workload and personnel staffing at night [39]. Doctors’ well-being may also be an explaining factor. For example, doctors from *Engaged* and *Mindful* may experience work engagement, while those from *Indifferent* and *Lethargic* may experience less work engagement and potentially even signs of burnout. Burnout may undermine the quality of care provided and is defined by emotional exhaustion, depersonalization, and reduced personal accomplishment [40]. These terms are akin to descriptors used in this study, such as mentally slow, withdrawn, and incompetent. A recent study identified different burnout phenotypes among surgery trainees and showed that burnout can be complex and have variable manifestations, for which different interventions may be needed [30].

Although more research is needed, we can speculate about the consequences of falling into a specific profile. Here we consider the post-shift profiles. Doctors from *Lethargic* were characterised by relatively low scores on all outcome measures. Due to reduced alertness, they may be at risk of impaired cognitive and psychomotor performance, making medical errors, or being involved in vehicle crashes [1, 4, 41]. They may communicate and collaborate worse with colleagues due to low levels of contentedness and calmness, indicative of low levels of well-being[19]. After the night shift, this may pose particular risks to the quality of handoffs as researchers have shown handoffs consist of various cognitive tasks that require good communication [42]. Doctors in *Tired but satisfied* had the lowest levels of alertness and may also be at risk of the abovementioned consequences. However, they seem less prone to well-being issues given their contentedness levels. The *Tired but satisfied* may overestimate their alertness as they performed the most consecutive shifts and felt relatively well. Ganesan et al. showed that healthcare workers perceive themselves as less alert on the first night than during subsequent nights, although their objective performance was equally impaired [22]. The *Excited* group of doctors seem alert and contented but not calm. Sport research has shown that high levels of arousal (i.e. excitement) can be detrimental for tasks involving complex and fine controlled movements, such as archery and golf putting [43, 44]. Doctors’ excitement may hinder their performance in high complex tasks under stressful circumstances, such as surgery. In contrast, doctors classified as *Mindful* may be vigilant and calm which makes them able to concentrate and oversee the situation. Mindfulness has been related to various positive outcomes such as attention, ability to establish good relationships with colleagues and patients and psychological well-being in general [45, 46].

Our findings do not tell precisely why doctors belonged to a specific profile, nor the consequences for their professional performance after the night shift. If we would better understand the causes and consequences of falling into and changing to a specific profile, interventions to reduce the adverse effects of night shifts may be tailored. For example, doctors belonging to the *Indifferent* or *Lethargic* profile show relatively low alertness, contentedness, and calmness before and after the night shift. Therefore, doctors from both groups may benefit from interventions to reduce burnout, which are usually divided into individual- (e.g. attention to self-care) and organisation-directed (e.g. support flexible work schedules) interventions [47, 48]. Literature suggests that an aligned combination of both is most effective [47, 48]. Doctors belonging to *Ready* or *Tired but satisfied* may benefit from interventions training them to become more engaged in their work or retain their alertness levels. For the latter, researchers have proposed various practical interventions such as naps in combination with caffeine intake and adjusted workplace lighting [49–51]. If the *Tired but satisfied* tend to overestimate their performance, self-awareness training may also be beneficial. Doctors in *Excited* may use relaxation techniques to reduce arousal in stressful situations. Those in *Mindfu*l might want use of flexible working arrangements, enabling them to perform some post-shift activities.

The found associations between night shift circumstances and doctors’ alertness, contentedness and calmness suggest that for some doctors restricting duty hours may be beneficial for how they feel and think they perform at night [9, 13, 21]. At the same time, this study's findings indicate heterogeneity among doctors' perceptions of their night shift work. Other studies show that general measurements, such as duty hour restrictions, are not consistently associated with improving doctors' well-being or patient outcomes [14, 52]. More flexible or personalized approaches are needed to balance doctors' training needs, well-being, and performance [14, 52] – for which this study may provide some first insights.

### Strengths and limitations

A strength is that this study provided a more nuanced insight into doctors’ alertness, contentedness and calmness before and after night shifts than previously known by identifying doctor profiles. A limitation of this study is that the pre- and post-shift profiles were calculated on unequal samples, which may have influenced the obtained profile solution. Nonetheless, the samples overlap, and our results showed no substantial differences in doctors' demographics. Although we used a diverse sample including trainees and consultants from the specialties surgery and OB/GYN, it is unknown whether the profiles can be generalised to other populations.

Another strength of this study was that we used validated measures to assess clinical fitness after performing night shifts with relevant reference values [27, 28]. A potential downside of the used scale is that it has been mainly used in the context of substance use, such as caffeine intake [53], and not so much in the context of occupational performance and well-being. Contrarily, the visual analogue scales used may be more feasible and better detect subtle before-after changes than often used instruments in such contexts with Likert-type scales [27, 54].

While this study included various variables about night shift circumstances, a limitation is that we could not fully explain the identified profiles and assess the consequences in terms of doctors’ professional performance after the night shift.

### Recommendations for research and practice

This study identified various doctor profiles based on their alertness, contentedness, and alertness. Future research could replicate our findings using larger samples, perhaps aimed at specific subgroups (e.g. surgery trainees). It would also be relevant to study whether profiles stay stable over multiple night shifts, or whether the representation of profiles varies when multiple consecutive night shifts are investigated. Moreover, studies could investigate the causes of belonging to a particular profile and the consequences to doctors’ professional performance after the night shift. For example, researchers may investigate how doctors from different profiles perform handovers after a night shift.

For practice, this study suggests that healthcare organisations should consider investing in safeguarding doctors working at night, and thus protect their patients. In general, healthcare organisations should aim to identify doctors at risk of impaired performance and well-being timely. While healthcare organisations may also cautiously investigate flexible working arrangements for doctors, they should aim to establish healthy workplaces. For example, by addressing the six areas of the worklife model: workload, control, reward, community, fairness, and values [55]. Doctors themselves could participate in interventions to improve their well-being, self-awareness or alertness.

## Conclusions

This study sought to obtain more nuanced insight into how doctors feel before and after night shifts to further attenuate the adverse effects of night shifts. RQ1 focused on identifying profiles based on doctors' self-rated alertness, contentedness, and calmness. The analysis identified three pre-shift (*Indifferent*, *Ready*, *Engaged*) and four-post shift profiles (*Lethargic*, *Tired but Satisfied*, *Excited*, *Mindful*). RQ 2 investigated changes in doctors' pre- and post-shift profiles and respective differences in alertness, contentedness and calmness scores. The results showed that most doctors change from Ready to Tired but Satisfied, with the most substantial reductions in alertness. Responding to RQ 3, we found that sleep and the number of consecutive shifts were the most important night shift circumstances predicting alertness, contentedness and calmness change. Future research may build on these findings by investigating the causes—including factors outside work—and the consequences of belonging to a particular profile regarding doctors' professional performance as well as whether specific subgroups benefit from tailored interventions.

### Supplementary Information

**Additional file 1:** Additional materials. Questionairres used and mean differences in observed profile changes.

## Data Availability

The data that support the findings of this study are available on request from the corresponding author. The data are not publicly available due to privacy or ethical restrictions.

## References

[1] Barger LK, Cade BE, Ayas NT, Cronin JW, Rosner B, Speizer FE (2005). Extended work shifts and the risk of motor vehicle crashes among interns. N Engl J Med.

[2] Rivera AS, Akanbi M, O’Dwyer LC, McHugh M (2020). Shift work and long work hours and their association with chronic health conditions: a systematic review of systematic reviews with meta-analyses. PLoS ONE.

[3] Wickwire EM, Geiger-Brown J, Scharf SM, Drake CL (2017). Shift work and shift work sleep disorder: clinical and organizational perspectives. Chest.

[4] Mansukhani MP, Kolla BP, Surani S, Varon J, Ramar K (2012). Sleep deprivation in resident physicians, work hour limitations, and related outcomes: a systematic review of the literature. Postgrad Med.

[5] Trockel MT, Menon NK, Rowe SG, Stewart MT, Smith R, Lu M (2020). Assessment of physician sleep and wellness, burnout, and clinically significant medical errors. JAMA Netw Open.

[6] Dula DJ, Dula NL, Hamrick C, Wood GC (2001). The effect of working serial night shifts on the cognitive functioning of emergency physicians. Ann Emerg Med.

[7] Maltese F, Adda M, Bablon A, Hraeich S, Guervilly C, Lehingue S (2016). Night shift decreases cognitive performance of ICU physicians. Intensive Care Med.

[8] Rollinson DC, Rathlev NK, Moss M, Killiany R, Sassower KC, Auerbach S (2003). The effects of consecutive night shifts on neuropsychological performance of interns in the emergency department: a pilot study. Ann Emerg Med.

[9] Wali SO (2011). Effects of on-call shifts on physicians’ cognitive performance, level of alertness, mood, and safety: a review article. Saudi J Internal Med..

[10] Wesnes KA, Walker MB, Walker LG, Heys SD, White L, Warren R (1997). Cognitive performance and mood after a weekend on call in a surgical unit. Br J Surg.

[11] Huizinga CRH, Tummers FHMP, Marang-van de Mheen PJ, Cohen AF, van der Bogt KEA (2019). A review of current approaches for evaluating impaired performance in around-the-clock medical professionals. Sleep Med Rev.

[12] Landrigan CP, Rothschild JM, Cronin JW, Kaushal R, Burdick E, Katz JT (2004). Effect of reducing interns' work hours on serious medical errors in intensive care units. N Engl J Med.

[13] Weaver MD, Landrigan CP, Sullivan JP, O'Brien CS, Qadri S, Viyaran N (2020). The association between resident physician work-hour regulations and physician safety and health. Am J Med.

[14] Awan M, Zagales I, McKenney M, Kinslow K, Elkbuli A (2021). ACGME 2011 duty hours restrictions and their effects on surgical residency training and patients outcomes: a systematic review. J Surg Educ.

[15] Antiel RM, Reed DA, Van Arendonk KJ, Wightman SC, Hall DE, Porterfield JR (2013). Effects of duty hour restrictions on core competencies, education, quality of life, and burnout among general surgery interns. JAMA Surg.

[16] Schwarz N (2000). Emotion, cognition, and decision making. Cogn Emot.

[17] Verster JC, Roth T (2012). Drivers can poorly predict their own driving impairment: a comparison between measurements of subjective and objective driving quality. Psychopharmacology.

[18] Kushnir T, Kushnir J, Sarel A, Cohen AH (2010). Exploring physician perceptions of the impact of emotions on behaviour during interactions with patients. Fam Pract.

[19] Desmet PMA (2015). Design for mood: twenty activity-based opportunities to design for mood regulation. Int J Des.

[20] Costa C, Mondello S, Micali E, Indelicato G, Licciardello AA, Vitale E (2020). Night shift work in resident physicians: does it affect mood states and cognitive levels?. J Affect Disord.

[21] Wali S, Qutah K, Abushanab L, Basamh RSA, Abushanab J, Krayem A (2013). Effect of on-call-related sleep deprivation on physicians' mood and alertness. Ann Thorac Med..

[22] Ganesan S, Magee M, Stone JE, Mulhall MD, Collins A, Howard ME (2019). The impact of shift work on sleep, alertness and performance in healthcare workers. Sci Rep.

[23] Lockley SW, Cronin JW, Evans EE, Cade BE, Lee CJ, Landrigan CP (2004). Effect of reducing interns' weekly work hours on sleep and attentional failures. N Engl J Med.

[24] Kusurkar RA, Mak-van der Vossen M, Kors J, Grijpma J-W, van der Burgt SME, Koster AS (2021). ‘One size does not fit all’: the value of person-centred analysis in health professions education research. Perspect Med Educ..

[25] Tummers FHMP, Huizinga CRH, van Pampus MG, Stockmann HBAC, Cohen AF, van der Bogt KEA (2021). Assessment of fitness to perform using a validated self-test in obstetric and gynecological night shifts in the Netherlands. Am J Obstetr Gynecol..

[26] Tummers FHMP, Huizinga CRH, Stockmann HBAC, Hamming JF, Cohen AF, van der Bogt KEA (2019). Objective assessment of fitness to perform (FTOP) after surgical night shifts in the Netherlands: an observational study using the validated FTOP self-test in daily surgical practice. Ann Surg.

[27] Bond A, Lader M (1974). The use of analogue scales in rating subjective feelings. Br J Med Psychol.

[28] Huizinga CRH, de Kam ML, Stockmann HBAC, van Gerven JMA, Cohen AF, van der Bogt KEA (2018). Evaluating fitness to perform in surgical residents after night shifts and alcohol intoxication: the development of a “fit-to-perform” test. J Surg Educ.

[29] Muthén B, Muthén LK (2000). Integrating person-centered and variable-centered analyses: growth mixture modeling with latent trajectory classes. Alcoholism: Clin Exp Res..

[30] Huang R, Hewitt DB, Cheung EO, Agarwal G, Etkin CD, Smink DS (2021). Burnout phenotypes among US general surgery residents. J Surg Educ..

[31] Mak-van der Vossen MC, van Mook WNKA, Kors JM, van Wieringen WN, Peerdeman SM, Croiset G (2016). Distinguishing three unprofessional behavior profiles of medical students using latent class analysis. Acad Med..

[32] Silkens MEWM, Chahine S, Lombarts KMJMH, Arah OA (2018). From good to excellent: improving clinical departments’ learning climate in residency training. Med Teach.

[33] Nylund KL, Asparouhov T, Muthén BO (2007). Deciding on the number of classes in latent class analysis and growth mixture modeling: a monte carlo simulation study. Struct Equ Modeling.

[34] Cohen J. The effect size. Statistical power analysis for the behavioral sciences. 2nd edn. Erlbaum: Hillsdale, MI, 1988; ISBN 0-8058-0283-5.

[35] Fey CF, Hu T, Delios A (2023). The measurement and communication of effect sizes in management research. Manag Organ Rev.

[36] Cygler J, Page AV, Ginsburg S (2021). Life on call: perspectives of junior and senior internal medicine residents. Acad Med.

[37] Storemark SS, Fossum IN, Bjorvatn B, Moen BE, Flo E, Pallesen S (2013). Personality factors predict sleep-related shift work tolerance in different shifts at 2-year follow-up: a prospective study. BMJ Open.

[38] Webb JM, Fu Y-H (2021). Recent advances in sleep genetics. Curr Opin Neurobiol.

[39] Mazur LM, Mosaly PR, Hoyle LM, Jones EL, Marks LB (2013). Subjective and objective quantification of physician’s workload and performance during radiation therapy planning tasks. Pract Radiat Oncol.

[40] Maslach C, Schaufeli WB, Leiter MP (2001). Job burnout. Annu Rev Psychol.

[41] Rosekind MR (2005). Underestimating the societal costs of impaired alertness: safety, health and productivity risks. Sleep Med.

[42] Militello LG, Rattray NA, Flanagan ME, Franks Z, Rehman S, Gordon HS (2018). “Workin' on our night moves”: how residents prepare for shift handoffs. Jt Comm J Qual Patient Saf.

[43] Oxendine JB (1970). Emotional arousal and motor performance. Quest.

[44] Janelle CM, Fawver BJ, Beatty GF. Emotion and sport performance. Handbook of Sport Psychology2020. p. 254–98. 10.1002/9781119568124.ch13.

[45] Scheepers RA, Emke H, Epstein RM, Lombarts KMJMH (2020). The impact of mindfulness-based interventions on doctors’ well-being and performance: a systematic review. Med Educ.

[46] Eberth J, Sedlmeier P (2012). The effects of mindfulness meditation: a meta-analysis. Mindfulness.

[47] West CP, Dyrbye LN, Erwin PJ, Shanafelt TD (2016). Interventions to prevent and reduce physician burnout: a systematic review and meta-analysis. Lancet.

[48] Panagioti M, Panagopoulou E, Bower P, Lewith G, Kontopantelis E, Chew-Graham C (2017). Controlled interventions to reduce burnout in physicians: a systematic review and meta-analysis. JAMA Intern Med.

[49] Schweitzer PK, Randazzo AC, Stone K, Erman M, Walsh JK (2006). Laboratory and field studies of naps and caffeine as practical countermeasures for sleep-wake problems associated with night work. Sleep.

[50] Smith MR, Fogg LF, Eastman CI (2009). Practical interventions to promote circadian adaptation to permanent night shift work: study 4. J Biol Rhythms.

[51] Song Y, Lv X, Qin W, Dang W, Chen Z, Nie J (2021). The effect of blue-enriched white light on cognitive performances and sleepiness of simulated shift workers: a randomized controlled trial. J Occup Environ Med.

[52] Ahmed N, Devitt KS, Keshet I, Spicer J, Imrie K, Feldman L (2014). A systematic review of the effects of resident duty hour restrictions in surgery: impact on resident wellness, training, and patient outcomes. Ann Surg.

[53] Haskell CF, Kennedy DO, Wesnes KA, Scholey AB (2005). Cognitive and mood improvements of caffeine in habitual consumers and habitual non-consumers of caffeine. Psychopharmacology.

[54] Cummins RA, Gullone E, editors. Why we should not use 5-point Likert scales: the case for subjective quality of life measurement. Proceedings, second international conference on quality of life in cities; 2000; 74(2): 74–93.

[55] Montgomery A, Panagopoulou E, Esmail A, Richards T, Maslach C (2019). Burnout in healthcare: the case for organisational change. BMJ.

