# Supplementary material for: Doctors’ alertness, contentedness and calmness before and after night shifts: a latent profile analysis
Source: Hum Resour Health. 2023 Aug 21;21:68. doi: 10.1186/s12960-023-00855-z (PMC10441714; doi:10.1186/s12960-023-00855-z)
Supplement: Supplementary file 1 — Additional file 1: Additional materials. Questionairres used and mean differences in observed profile changes. [file 12960_2023_855_MOESM1_ESM.docx]

**Additional materials**

A) The 16 items representing the Bond and Lader Visual Analogue Mood Rating Answer Scales*

VASBL01 Alert – Drowsy

VASBL02 Calm – Excited

VASBL03 Strong – Feeble

VASBL04 Confused – Clear-headed

VASBL05 Well-coordinated – Clumsy

VASBL06 Lethargic – Energetic

VASBL07 Contented – Discontented

VASBL08 Troubled – Tranquil

VASBL09 Mentally slow – Quick-Witted

VASBL10 Tense – Relaxed

VASBL11 Attentive – Dreamy

VASBL12 Incompetent – Proficient

VASBL13 Happy – Sad

VASBL14 Antagonistic – Amicable

VASBL15 Interested – Bored

VASBL16 Withdrawn – Gregarious

*Each item is rated using a Visual Analogue Scale.

Calculation of each of the three domains using the 16 individual items represented above.

**VAS Alertness**= 100-((VASBL01)+(VASBL03)+100-(VASBL04)+(VASBL05)+100-(VASBL06)+100- (VASBL09)+(VASBL11) +100-(VASBL12)+(VASBL15)) / 9
**VAS Contentedness**= 100-((VASBL07)+100-(VASBL08)+(VASBL13)+100-(VASBL14)+100-(VASBL16)) / 5
**VAS Calmness=** 100- ((VASBL02)+100-(VASBL10)) / 2

B) Fit to Perform Questionnaire

*Personal and experience data*

- Function
  - Attending/ resident (AIOS or ANIOS)
- In case of an attending: subspecialty
- Man/Woman
- Years of experience
  - In case of attending
    - Year of graduation residency
  - In case of resident
    - Year of graduation medical doctor
- Partnership/Salaried Employment
- Hours sleep per night (estimated mean last 4 weeks)
  - Numeric
- Hours work per week (estimated mean last 4 weeks)
  - Numeric

*Shift characteristics*

- Type of measurement
  - Non-call measurement/ precall/postcall
- Do you qualify yourself as fit to perform surgery
  - Yes/No
- Do you qualify yourself as fit to see patients at the outpatient clinic
  - Yes/No
- Would you prefer to move a planned surgery, or let someone else perform the surgery?
  - Yes/No
- In case of Non-call measurement
  - Hours slept
    - Numeric
- In case of precall
  - Hours already worked
    - Numeric
- In case of postcall
  - Total hours of shift
    - Numeric
  - Number of consecutive nightshift
    - Numeric
  - Hours awake
    - Numeric
  - Hours slept
    - Numeric
  - Percentage activity during shift (calls/OR/ER or ward/nothing)
    - Numeric, total max of 100%
  - Do you need to continue work activities?
    - Yes/no

C) Table: *Delta mean alertness, contentedness and calmness scores for observed change pattern*

| Profile change pre - post | N= | ΔM±SD Alert. | ΔM±SD Content. | ΔM±SD Calm. |
| --- | --- | --- | --- | --- |
| Indifferent - Lethargic | 9 | -4.05±6.14 | -4.42±5.37 | -4.17±4.56 |
| Indifferent - Tired but satisfied | 12 | -9.90±11.1 | +1.03±7.32 | +10.4±16.7 |
| Ready - Lethargic | 3 | -21.9±9.28 | -12.7±17.6 | +9.17±19.8 |
| Ready - Tired but satisfied | 46 | -20.8±15.96 | -5.76±13.5 | +3.11±21.5 |
| Ready - Excited | 5 | -5.18±11.1 | -4.88±13.8 | -12.3±20.2 |
| Ready - Mindful | 10 | +11.4±12.5 | +11.6±10.4 | +16.4±11.8 |
| Engaged - Tired but satisfied | 23 | -22.9±12.0 | -10.2±12.2 | -11±11.6 |
| Engaged - Mindful | 27 | -2.55±6.46 | -0.48±6.18 | +0.28±6.62 |
